# Shared latrines in Maputo, Mozambique: exploring emotional well-being and psychosocial stress

**DOI:** 10.1186/s12914-018-0169-z

**Published:** 2018-07-25

**Authors:** Tess Shiras, Oliver Cumming, Joe Brown, Bacelar Muneme, Rassul Nala, Robert Dreibelbis

**Affiliations:** 1 Johns Hopkins Bloomberg School of Public Health, 615 Wolff Street, Baltimore, MD USA; 2 London School of Hygiene and Tropical Medicine, Department of Disease Control, Kepple Street, London, UK; 3 School of Civil and Environmental Engineering, Georgia Institute of Technology, Atlanta, GA USA; 4 WE Consult, Water Supply and Mapping, 1013 Ave. Kwame Nkrumah, Maputo, Mozambique; 5 Ministry of Health, Republic of Mozambique, Maputo, Mozambique

**Keywords:** Sanitation, Shared latrines, WASH, Psychosocial stress, Stress, Gender

## Abstract

**Background:**

Approximately 18% of Sub-Saharan Africa’s urban population relies on shared sanitation facilities, which are shared by one or more households. While there is growing recognition of sanitation’s relationship with stress and well-being – particularly among women – most research has focused on rural populations and the transition from open defecation and/or unimproved latrines to private shared sanitation. This study explores sanitation-related stressors among users of both improved and unimproved shared sanitation facilities.

**Methods:**

This study was nested within the larger MapSan health impact trial (Trial Registration: NCT02362932). Participants were recruited from the control arm of the trial (Traditional Latrine (TL) users) and intervention arm, which received one of two improved shared sanitation facilities – Shared Latrines (SL) shared by up to 20 individuals and Community Sanitation Blocks (CSBs) shared by more than 20 individuals. Sampling was informed by a life stage perspective to reflect diversity in sanitation needs and experiences within the population. Data included 96 in-depth interviews, 7 focus group discussions, and 25 unstructured observations. Data collection and analysis followed a Grounded Theory approach, which was used to identify the key domains of sanitation-related stress among participants. A semi-structured tool was applied to all female interview transcripts to assess the frequency and severity of key stressors.

**Results:**

Participants reported stress due to: lack of safety; lack of privacy; disgust about the latrine condition; and collective action failure in terms of managing the latrine, often causing neighborhood conflict or unhygienic sanitation conditions. Fewer SL and CSB users reported specific stress domains and – with the exception of perceived safety – reported fewer severe stressors. The leading cause of stress reduction due to the intervention was decreased disgust followed by increased privacy and safety.

**Conclusions:**

Our data suggest that “improved”, shared facilities can reduce stress when proper maintenance and management systems are in place. Private, shared sanitation only had limited impact on users’ perceptions of safety, particularly at night, suggesting that safety concerns extend beyond the physical latrine structure. Our research demonstrates that factors including latrine location and neighborhood violence are important determinants of safety perceptions and corresponding psychosocial stress.

## Background

Despite increased attention from global donors and governments to water, sanitation, and hygiene (WASH), 2.3 billion people across the world lack access to improved sanitation facilities [1]. Of this group, 600 million people use improved but shared latrines [1]. Rates of shared, improved sanitation are highest in urban sub-Saharan Africa, at 18% [1]. In Mozambique, 53% of urban residents lack access to improved sanitation, including approximately 9% of the population that uses shared, improved facilities [1]. According to the World Health Organization and UNICEF Joint Monitoring Programme (JMP), improved sanitation facilities shared between two or more households are defined as ‘limited’ sanitation services [1]. For the purposes of this manuscript, we refer to upgraded, ‘limited’ sanitation services as ‘improved’ shared latrines.

Negative stress – or distress – is the result of either real or perceived threats that exceed an organism’s capacity to successfully manage said threat. Psychosocial stress typically refers to the negative stressors that individuals experience as a result of the social (i.e., human) environment in which they operate. Prolonged psychosocial distress is associated with a number of negative health outcomes, including: mood and anxiety disorders [2, 3], poor cardiovascular health [4], onset and aggravation of diabetes [5], negative lifestyle changes such as smoking and under or over eating [6] and decreased quality of life. Several studies have found that poor sanitation is associated with psychological distress among women [7–15]. Studies in Indonesia [13], India [16], South Africa [17], Uganda [18], and Kenya [19] have found that inadequate sanitation contributes to psychosocial stress through experiences and fear of sexual and physical violence, feeling unsafe when using latrines at night, and shame and embarrassment due to lack of privacy.

At a policy level, there is growing recognition that effective, equitable, and sustainable sanitation interventions need to consider the associated psychosocial vulnerability among girls and women [1, 20–23]. However, there has been relatively little research to understand female user experiences with sanitation, particularly in regards to stress, vulnerability, and dignity in different contexts and geographic areas [13, 18, 23, 24]. Existing literature on WASH and psychosocial stress has primarily focused on women in rural populations undergoing the transition away from unimproved latrines or open defecation to private improved latrines [7, 11, 16]. The impact of improved shared latrines, in contrast to unimproved shared latrines, in urban areas on psychosocial stress and emotional well-being remains unexplored [16, 14, 13, 25]. Understanding the impact of shared sanitation interventions on stress and well-being can inform the development of such interventions to increase gender equality, address female empowerment with respect to sanitation access and use, and contribute to sustained use and maintenance of facilities.

The aim of this study is to investigate whether, and how, improved shared latrines in informal urban settings can reduce sanitation-related stressors and improve women’s well-being. This research used qualitative methods to examine access, use, and maintenance patterns between users of unimproved and improved shared sanitation facilities in peri-urban informal settlements in Maputo, Mozambique. The research also explored women’s attitudes towards and satisfaction with sanitation options. This study was conducted in the context of an intervention, which allowed comparison between experiences of users of improved and unimproved shared facilities as well as within individual intervention beneficiaries who had moved from using an unimproved to an improved shared latrine in the past year. Of particular interest for our investigation was identifying the specific domains of sanitation-related stress, the factors that influenced both the frequency and severity of these stressors, if stressors differed between traditional and improved shared latrine users, and – within the context of the larger MapSan intervention – if stressors differed between different types of improved sanitation users.

## Methods

### Study site – Maputo, Mozambique

Maputo has a population of approximately 1.2 million people, 70% of whom live in informal slum settlements [26]. Migration to Maputo increased significantly during the war for independence (1964–1974) and subsequent civil war (1974–1992), as people sought refuge from the conflicts but also sought economic opportunities [26]. Currently, Maputo has a mean population density of 4086 people per square kilometer, although this varies significantly across neighbourhoods. Between now and 2050, approximately two-thirds of the country’s population growth is expected to occur in cities, a trend that will put additional stress on already inadequate public health infrastructure and further contribute to the “urbanization of poverty” [26]. An estimated 89% of all Maputo residents use on-site fecal management systems (non-sewered), and only 26% of all fecal waste in the city is safely managed^1^ [28].

### Study site - intervention description and setting

This study was nested within the larger MapSan health impact trial, a controlled before-and-after (CBA) study to evaluate the effect of a sanitation intervention on child health (clinicaltrials.gov NCT02362932; Brown et al. [27]). Water and Sanitation for the Urban Poor (WSUP), an international development non-profit, implemented the shared sanitation intervention between March 2015 and March 2016. The intervention included construction of pour-flush toilets with a septic tank connected to an infiltration pit (some squat and some with a pedestal) that are shared among compound residents. These latrines have two variants: a Shared Latrine (SL) is a single latrine unit constructed with cement blocks and a metal door, designed for compounds with 20 or fewer residents. A Compound Sanitation Block (CSB) uses similar construction but is designed for compounds with more than 20 residents, with a cubicle for every 20 people (images included in Supplementary Materials). The CSBs have a piped water connection with a water storage tank and tap on the outside of the latrine (although residents have to organize and pay for water supply access), a rain harvesting storage tank connected to a water tap inside the latrine, a clothes-washing station, and one handicapped-accessible unit. Both SLs and CSBs have a drain and a sink basin. In contrast to the CSBs, the SLs do not have a piped water connection. WSUP implemented the intervention in 11 *bairros* (neighborhoods) in peri-urban informal slum settlements in Maputo City, and this research study identified participants from compounds within these same 11 *bairros*. Control compounds using unimproved shared sanitation were from these same 11 *bairros* plus an additional 5 *bairros* adjacent to them.

In these *bairros*, groups of small houses—made from wood, corrugated iron sheets, or concrete—are clustered together around a small, multi-purpose shared space used for cooking, cleaning, playing, and working. Each set of houses and its shared space is called a *compound* and is often delineated by a wall or fence. Approximately 20% of all households in informal neighborhoods of the city are organized as compounds, typically prevalent among the poorest families. The WSUP-constructed latrines are situated within each compound’s shared space, with the specific location determined by engineering considerations and site constraints. Thus, the latrine position within the compound varies: it could stand near the entrance, extremely close to one, or several, homes, in a corner, or more centrally located in the shared space and further from individual houses. Traditional latrines may be found inside or just outside of a compound’s defined space. Compounds are connected to one another by several car-accessible dirt roads but primarily by narrow, winding pathways. A compound’s shared space is not considered public space: access to latrines built in common areas is generally limited to residents of the compound.

### Sample & participant selection

Our sampling frame was the control and intervention compounds of the MapSan trial. We purposively sampled three compound types based on the type of sanitation facility: 1) compounds sharing a traditional (pit) latrine^2^ (control group), 2) MapSan intervention compounds that received and share a SL (one-unit WSUP improved latrine), and 3) MapSan intervention compounds that received and share a CSB (the larger WSUP improved latrine).

Sampling was informed by a life stage perspective that organizes life events around specific biologically and socially defined periods. We purposively selected participants that met the following criteria: individuals with children under 3 years, individuals whose youngest child was between three and 18 years, individuals with no children or whose youngest child was older than 18 years, individuals who married in the past 2 years, and individuals who moved to the compound in the last 2 years. These life stages are not mutually exclusive; if respondents met more than one criteria, they were included in all groups. However, if respondents had children in multiple age categories, they were included in the group with their youngest child, as younger children require more latrine-related assistance than older children and this better represented the respondent’s sanitation responsibilities. These stages reflect life changes that may alter individuals’ use of, access to, and attitudes towards sanitation facilities. The study sample aimed to include 75% women and 25% men, as women are responsible for the majority of sanitation-related tasks and are likely to spend more time in the compound given economic opportunities [29].

### Data collection

The study used qualitative methods to investigate psychosocial stressors, emotional well-being, and user satisfaction among users of shared improved and shared traditional latrines. A five-person data team was trained in qualitative methods including purposive sampling, semi-structured in-depth interview techniques and probing, focus group moderation, and unstructured observation techniques. The team conducted 96 in-depth interviews (IDIs), seven focus group discussions (FGDs), and 25 unstructured observations. We adapted research guides on an iterative basis as new themes emerged and as data collectors reported on lessons learned in the field. We terminated sampling once the research team determined that the data had reached theoretical saturation, the point at which themes are repeated and no new information appears in the data. Observation sample sizes varied from 6 to 60 people, as this sample size was based on the catchment areas of the compound unit. Each FGD had a range of 5–12 people, summing a total of 47 participants. Some individuals who participated in a focus group also participated in an IDI and/or observation.

### Data analysis

All interviews and focus groups were conducted in the preferred language of the participant which was either Portuguese or Xichangana, audio recorded, transcribed verbatim, and translated into English. The data team provided detailed summaries of unstructured observations including a chronology of observed activities. Data were analyzed concurrent to data collection. Methods were informed by Grounded Theory [30]: the study team did not use an existing theoretical framework or set of assumptions to guide analysis. Rather, the team analyzed data inductively by reading transcripts, summarizing data collection events, holding frequent team debriefs, and conducting initial line-by-line coding to identify relevant themes. This process informed the development of thematic memos and development of an analytic codebook, which was used to code all data with *NVivo* software.

Interview transcripts were also analyzed using a structured stress-level questionnaire to better understand the magnitude and frequency of reported stressful experiences. This questionnaire used a categorical scale (not present, mild, moderate, severe) to analyze distinct sanitation-related psychosocial stressors identified during preliminary codebook development. This approach provided insight into the relationship between the severity of specific stressors and their corresponding frequency (never, sometime, often) as well as differences in stress severity, frequency, and causes between intervention and non-intervention groups. As data collection was not guided by an existing theoretical framework or assumptions about possible stressors, each interview included discussion of different types of stressors—or possibly of no stressors at all—dependent on the flow of the interview and the respondent’s unique sanitation experiences. Thus, the stress-level questionnaire was limited only to those respondents that either spontaneously provided information on a specific stressor or who responded to a direct inquiry.

Collecting and analyzing data simultaneously allowed the research coordinator to continuously adapt the codebook, data collection guides, and sampling frames as new themes and lessons from the field emerged.

### Ethical approval

All interview and focus group participants provided written informed consent for both data collection and publication prior to data collection. All interview and focus group participants were at least 18 years of age. Data collectors informed participants of their right to end the data collection event at any time or skip any questions. Compound chiefs were provided with and signed a participant information sheet prior to observations but did not sign a written consent form, as observations occurred in public spaces. No personally identifiable information was collected and any names or other identifiers included in audio recordings were redacted during transcription. All audio recordings were permanently deleted.

This study received approval from the Ethics Committee of the London School of Hygiene and Tropical Medicine (Ref: 11791) and from the Republica De Moçambique Ministério da Saúde Comité Nacional de Bioética para a Saúde (IRB00002657) prior to inception of research activities.

## Results

### Participant characteristics

The data team collected 96 interviews, 70 with women (73%) and 26 with men (27%). Given the gendered impacts of sanitation-related psychosocial stress, this analysis draws primarily on data collected from female participants. Of the 70 IDIs with women, 22 (31%) used a SL, 29 (42%) used a CSB, and 19 (27%) used a traditional latrine. Table 1 below shows demographic characteristics for female IDI participants in total and by intervention and control groups.

**Table 1:** Demographic characteristics of female IDI respondents using shared sanitation by latrine type

Demographic Characteristic	Total *n* = 70% (n) or Mean (range)	Shared latrine (SL) *n* = 22% (n) or Mean (range)	Compound sanitation block (CSB) *n* = 29% (n) or Mean (range)	Control (i.e., traditional latrine) *n* = 19% (n) or Mean (range)
Life Stage				
Single	22% (15)	23% (5)	21% (6)	26% (5)
Married	54% (37)	45% (10)	52% (15)	68% (13)
Separated	6% (4)	14% (3)	3% (1)	0% (0)
Widow	18% (12)	18% (4)	24% (7)	6% (1)
Married within last 2 years	16% (11)	9% (2)	17% (5)	21% (4)
Age	35 (19–70)	37 (20–65)	37 (19–70)	37 (22–60)
# Children	2.2 (0–6)	2 (0–5)	2.3 (0–6)	2.2 (0–6)
Children < 3 years	49% (34)	55% (12)	55% (16)	32% (6)
Children 3–18 years	59% (41)	59% (13)	52% (15)	68% (19)
New to compound	23% (16)	23% (5)	21% (6)	26% (5)
Education Level	6 (0–12)	7 (0–12)	5 (0–11)	6 (0–12)
Mean # HHs sharing	11 (1–100)	5 (1–9)	19 (2–100)	7 (2–22)

### Sanitation-related psychosocial distress causes

Four primary sources of sanitation-related distress were identified in our data: lack of safety; lack of privacy; feelings of disgust or shame about the condition of the latrine; and collective action failure in terms of managing the latrine, often leading to conflict among users or unhygienic sanitation conditions. While the research team expected to identify stress due to the first three identified domains, stress caused by management processes—or lack thereof—was an unexpected theme that emerged during data analysis.

#### Safety and security

Over half (68%) of all female participants mentioned safety concerns related to sanitation use, primarily due to fear of robbery or physical and sexual assault. Data suggest that improved shared latrines, particularly SLs, reduced the frequency of distress related to safety and security. Less than half (41%) of SL users reported safety and security stressors compared to approximately 80% of both CSB and TL users. Approximately 80% of those identified as having safety concerns were categorized as having moderate or severe safety-related stress. In contrast to the frequency of stressors, the proportion of users with safety or security concerns categorized as moderate or severe was similar among all intervention groups (TL: 92%, SL: 88%, CSB: 73%). Further, of the 25 intervention users that compared feelings of safety in their traditional and improved latrine, 16 (64%) reported that their security had improved due to the new latrine.

*It was very risky…First because we have no yard and we had to go out [of the compound] to enter in the bathroom. Sometimes people, thieves entered stole, we were afraid sometimes of people beating us back there.* – CSB user comparing TL positioned outside the compound to the intervention CSB

*Without doubt, the new one is better in every way, excellent construction and my life improved… When I go to clean garbage I return home very late, I can warm my water to take a bath without a problem because I can lock the door during my bath safely.* – CSB User

*Before this bathroom, we had a precarious one, it was constructed of tires and stones… So when this bathroom was constructed, we had many benefits because now we can wash the bathroom, we have energy, we feel safe, because we have a door, now we can take a bath at night…Inside and outside, we can lock the latrine.* – SL User

Generally, female respondents felt that their communities were unsafe, particularly at night.

*I’m scared to go alone to the bathroom, I ask my husband to go with me because here in Xipamanine there is much violence and many robbers, we do not have security in the backyard and the bathroom is also in front of the street, if someone wants to hurt you he can do it even if you have keys locking the door.* - CSB User

One CSB participant reported an instance of attempted sexual assault while she was using the latrine, and several others mentioned hearing of similar attacks, making it unsafe to access the bathroom, especially at night. Robbery and theft were also common risks faced at night:

*I fear because of the way that the bathroom is, you think about going out for the bathroom, while there may be a hidden person who can beat or kill you, rape, so many things that happen around here.* – TL User

Lack of lighting and lack of fencing compounded safety concerns. About one third of participants (both improved and traditional latrines users) in IDIs and all women in a focus group with SL users reported urinating in a bucket at nighttime due to fear of “bandits” (“*bandidos*”). Most of these women commented that needing to defecate is the only instance in which they leave the house during the nighttime. Although urinating in a bucket at night was reported among users both with and without secure fencing, several women specifically noted that lack of compound walls made them feel more insecure.

The sense of security among improved latrine users increased due to better constructed bathrooms, the presence of doors, locks on the inside and outside of the doors, and the proximity of the new latrine inside their compound walls. Improvements were not uniform among all intervention beneficiaries.

#### Privacy and embarrassment

Participants who experienced privacy-related stressors complained of the poorly-constructed latrine infrastructure and the placement of the latrine close by to communal areas. Among all respondents, approximately one third (37%) reported stress related to privacy. Lack of latrine doors, walls, or roofs often allowed other community members to accidentally or purposefully see inside the latrine, while women were using it. This led to embarrassment and consistent stress.

Experiences of lack of privacy differed by latrine type. Approximately four out of every five TL respondents reported privacy-related stress while only one quarter of intervention respondents reported privacy-related stress (SL: 33%, CSB: 18%). Roughly 90% of privacy-related stress among TL users was categorized as moderate or severe while more than half of both SL and CSB reporting privacy stressors were categorized as mild. Further, all CSB users who compared their privacy experience pre- and post-improved latrine had decreased stress.

The majority of TL walls are made of reed or corrugated iron sheets, which often have holes or openings, allowing people to see inside while someone defecates or bathes. Additionally, TL users frequently reported someone entering while another is inside, all of which cause embarrassment.

*I don’t feel secure, but I don’t know where do I go to because actually I do not go to work… I live here as a prisoner because I don’t have a place to go to. There’s no door at the bathroom we only use iron to cross the door signaling when we are still using it. Even with a warning on the door there’s a risk of someone getting inside while you are using, then you apologize to each other.* – TL user

Locks were a major determinant of privacy-related stress. Traditional latrines often lack doors and locks are rare. Intervention latrines are always constructed with both doors and locks. At the time of data collection, approximately 80% of CSB users and 40% of SL users reported that their latrine door has a lock; this difference may explain the disparity in frequency of privacy-related stress between SL and CSB users.

Several participants also noted privacy concerns in cases when their latrine is located close to an individual household or to shared space where residents typically gather to sit and talk, making it easier for others to smell and hear defecation. In one case, a TL participant reported feeling so embarrassed that she would walk to her mother-in-law’s bathroom in a different compound if her husband or his friends were gathered close to the latrine. For some participants, even the act of entering the latrine in front of others was embarrassing or shameful, and respondents wished for a more private option.

*I don’t feel very good about [defecating] because I have fear of being seen when I am entering in the latrine to defecate. Everyone can see and know that I am defecating. Then I feel embarrassed when I leave and I see that someone is close by.* -TL User

This sense of shame was particularly notable among older females, who felt it was inappropriate to be seen moving to and from the bathroom.

*To go in toilet I had to take 2 or 3 capulanas (fabric) for cover, kids would be playing soccer. I am old, and it is shameful to be seen by children taking a bath, now we are happy with this toilet.* – CSB user on her previous experience with a traditional latrine

#### Latrine disgust

Disgust due to latrine conditions was noted among all user groups, although was far more frequent among TL users, all of whom reported experiencing disgust-related stress, compared to SL users (76%) and CSB users (69%). Further, all TL users were categorized as experiencing either moderate or severe disgust-related stress. Current TL respondents and intervention respondents reflecting on their old latrine noted revulsion, particularly when the pit latrine filled, clogged, or overflowed due to rain. TL participants also frequently complained of bad smells (especially when it was hot), the difficult process of removing feces and digging new pits (which occurs at least three to four times annually), and the visible presence of feces in and around the latrine.

*On hot days you can't sit down here, we have to stay inside of our houses with the door closed because of the bad smell of the latrine. As you see these houses are too small, keeping the door closed on the hot days is a sacrifice but we have no other choice…Before I came to live here my husband warned me that the latrine was horrible, but I didn't realize it was so bad.* – TL user

The majority of improved latrine SL and CSB users also reported feeling disgusted by their current latrine. However, roughly half of users that reported disgust-related stress in both intervention groups were categorized as mild (SL: 44%; CBS: 50%). All intervention users that compared their experience to their previous traditional latrine reported less disgust with the intervention bathroom.

*Before, this [old bathroom] here filled, the poop spread out here on the ground, sometimes it spread out until the poop was even where we cooked, as the space is very small. But it's different now, the bathroom has a very large space that can bathe two people, the toilet is also up high, we feel good with the bathroom.* – CSB user reflecting on old latrine

Eight respondents discussed how their previous or current TL could create diseases including diarrhea, cholera, and malaria (from mosquitoes at standing water). Similarly, intervention participants were relieved that the new latrine helped prevent disease while TL respondents expressed frustration about their pit latrine’s unhygienic conditions.

*Almost always we suffer from diarrhea and malaria, because that water creates many mosquitoes, and flies that contaminate our food and water are giving us diarrhea. We are always sick. Many of the women who have children here, when they go to the bathroom, they don’t put children’s feces into the shit-hole. They normally put it on the floor and then the flies come on it.* – TL user

Traditional latrine users also reported feeling embarrassment about the state of their latrine when a guest was present. Accordingly, several SL and CSB users noted that they could now welcome visitors into their home without feeling embarrassed if they needed to use the latrine.

*…the bathroom is a reflection of one’s home… this is to say, a lady that does not live here can come and ask to use the bathroom and if it is dirty, she can leave and say that in this house there are pigs only because people do not like washing the bathroom.* – CSB user

#### Management-related stress

Collective action occurs when a group of individuals have a ‘joint commitment’ to one another to perform a particular behavior together [31]. A collective action failure occurs in the absence of such a ‘joint commitment.’ When a particular activity requires action from multiple people rather than an individual, yet there is no joint consensus to perform the activity, a collective action failure occurs [31, 32]. The phenomenon of collective action failure as it relates to shared latrine cleaning and management has been discussed in literature by Tumwebaze and Mosler [33], in which they assert that social motivation is significantly associated with latrine cleaning behaviors. Without such social motivation, users often revert to an attitude of “why clean if others do not?” [33].

The majority of females in our study described poor latrine management as a collective action failure and a source of stress, exacerbating existing social tensions through compound gossip, neighborly disputes, and social exclusion. Management-related stress was most common among CSB and TL users (CSB: 77%,TL: 65%), while only about half of SL users – users who share latrines with far fewer households - discussed stress related to inadequate management. While over 70% of all users who reported on management-related stress were categorized as mild or moderate stressors, only four cases of management-caused stress were labeled as severe. For example, one CSB participant reported urinating, defecating, and bathing inside her home at all times to avoid harassment from her neighbors. She empties the urine and feces from her bucket into the latrine at strategic times when she does not think that anyone will see her. In another extreme case, a SL participant, who moved into the compound after the latrine had been constructed, has been denied access to the latrine by the compound head. This participant has attempted to solve this issue through mediated conversation, but she is forced to travel to her landlord’s latrine or use a bucket in her home.

More typically, participants complained in interviews about the lack of understanding and collective participation in latrine cleaning among compound members, leading to stress and disgust.

*My sister this compound has 4 latrines but only 1 is still in use, because the others are clogged. Even that one is not in [good] condition as I told you, but who cause a great problem is all of us because we don’t clean it. One thing is to be poor, it is another to be a pig, I think we could try to clean this bathroom even in the condition that it is.* – TL user

Some participants reported attempting to talk with those who left the latrine dirty or did not participate in cleaning. However, many interview respondents felt that they lacked agency to make changes related to the latrine or agency to influence others’ behavior. For example, many participants said that they did nothing to improve collective action due to “little understanding” among compound residents or feeling “tired of talking.” These women primarily felt that their neighbors would not change their behavior and that it was not worth their time or energy to continually discuss latrine maintenance with them.

Some respondents attempted to organize compound members to create cleaning schedules that would improve latrine hygiene conditions and mitigate disgust-related stress. However, many of these efforts were ineffective or unsustainable, resulting in collective action failure. Respondents that discussed management-related stress noted that they often had little agency in changing their latrine conditions. For example, participants expressed having dreams or a vision of an ideal latrine, but they lacked financial means to realize them. Additionally, several participants reported that they were tired of attempting to influence cleaning behaviors among their neighbors.

**Fig. 1:**
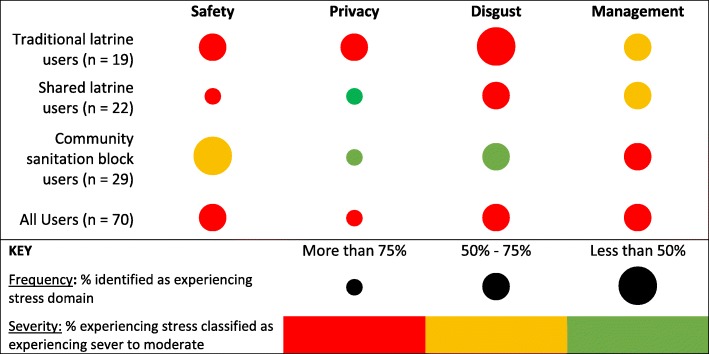
Frequency and severity of specific sanitation-related stress domains as identified in in-depth interviews with adult female respondents of shared sanitation in peri-urban Maputo, Mozambique]

Table 2 provides an overview of the frequency and severity of the major stress categories divided by intervention group. We used the total number of respondents per user group as the base population for percentage calculations. In most cases, individuals either discussed the issue unprompted or were directly questions about their experiences within each domain. However, we note that there are 1 to 3 respondents who were not asked directly about the specific stress domain or who did not discuss the domain spontaneously. In order to reflect this range of uncertainty, prevalence of stressors is presented as a range rather than specific percentage Fig. 1.

**Table 2:** Reported reduction in sanitation-related stress by intervention type among adult female in-depth interview participants

Compound Type	Any stress relief	High stress relief	Moderate stress relief	Low stress relief	No stress relief
Compound sanitation block (*n* = 25)*	92%	28%	48%	16%	8%
Shared latrine (*n* = 18)*	78%	17%	33%	28%	22%

*Note that sample size is slightly smaller, as it only includes respondents who compared their stress level before and after the intervention

While the domains of stress are presented here as unique factors, we note that they can mutually influence one another. The more complex management structures with CSBs may have resulted in more frequent management related stress. However, users from compounds in which residents sit together, vote on a leader, and implement latrine rules reported decreased feelings of disgust due to systematic cleaning. These systems may also result in improved operation and maintenance of facilities, as noted in the higher proportion of CSBs with locks compares to SLs, resulting in improved safety and privacy among users.

### Intervention impact on stress level

Participants in the intervention group – both SL and CSB users – reported experiencing fewer sanitation-related stressors than participants in the control group. Additionally, the majority of intervention respondents (89%) discussed how the new latrine lessened their stress-level. Over 50% of intervention respondents were categorized as having moderate or high stress relief due to the new latrines, while about 20% of respondents had low stress relief and another 20% reported no difference in stress levels between their current and previous latrine. CSB users were more likely to report reduced stress due to the intervention (92%) than SL users (78%). Further, a greater percentage of CSB users (76%) reported high to moderate stress relief due to the presence of the new latrine compared to 50% of SL users.

Intervention participants most frequently discussed reduction in their disgust-caused stress followed by privacy and then safety. Reductions in disgust also led to decreased feelings of embarrassment for some participants when presenting the bathroom to external visitors. Five participants mentioned that the new latrine decreased the possibility of diseases including diarrhea, cholera, and malaria. Reports of decreased disgust were approximately even across SL and CSB users. However, improved privacy was more frequently noted among CSB users than SL users. This may be because CSBs were more likely than SLs to have locks on the inside and outside of the latrine doors, and the larger CSB unit may be perceived as a more secure building. Conversely, more CSB users reported current safety concerns compared to SL users.

Management related stress did not decrease in intervention (SL and CSB) compared to control compounds. In fact, there was *increased* management-related stress among some CSB users. The CSBs, by definition, are shared by larger groups of people and have more complex management systems, including assigning users to specific latrines and having more formal and complex cleaning rosters. Reports of severe and moderate management-caused stress typically occurred in compounds with 30 or more people. However, this pattern does not always hold true: some individuals who live in compounds with 40–60 people reported no or only mild stress due to poorly managed latrines. Some CSB participants also explained that the new latrine requires more collective action to maintain and clean the facility. Thus, lack of proper cleaning is more evident in the newly constructed latrines compared to older-pit latrines, where unhygienic conditions persist as the norm.

### Stress among men

Sanitation-related stress is not confined to women. Male participants also discussed stressors such as disgust regarding the latrine condition, lack of privacy, and concern around fecal sludge management.

While females’ safety concerns were largely due to fear of thieves and assailants in the nighttime, male participants noted that thieves existed in the area but were not perceived as a threat to male participants. The majority of men reported that they felt secure using the latrine, even at night. In the all-male focus group discussion, when asked about security, participants agreed that the latrine was safe. Men were much more likely to discuss lack of privacy than safety issues when prompted about security or negative experiences with the latrine.

Our research found that men are typically tasked with fecal sludge management. Discussions of desludging focused on traditional latrines, as the large majority of intervention beneficiaries have not yet had to empty their septic tanks. Depending on the wealth of the compound, male residents either desludge on their own (for free or a small fee) or the hire informal workers from outside the compound to manually desludge and dig a new pit*.* In either scenario, emptying and rebuilding pits frequently caused stress among participants.

*What stresses me, too, is that the bathroom does not take long to fill. And we ourselves have to do the work of removal to another pit; that just stresses me out a lot. The ground is already saturated [with feces]. And it’s always filling and we dig where we have dug previously. Sometimes we just find stools that still get mixed with sand. It's very stressful.* – Male TL User

The desludging process for traditional latrines occurs frequently, about every 3 months, and can also be disruptive for neighbors due to the bad smell. Women occasionally remarked on desludging processes, as well. However, women’s reports were more factual and in passing whereas male participants remarked on the frustration and disgust they felt regarding their responsibilities to frequently desludge the traditional latrines. Although formal pit emptying services, which safely remove excreta from pits, are available in the community, these services were not mentioned during data collection.

## Discussion

The research team is unaware of other studies examining sanitation-related psychosocial stress within the context of an intervention that provides improved shared latrines. This unique perspective allowed for direct comparisons between users of traditional and improved shared latrines as well as within intervention beneficiaries who could compare their sanitation experiences pre and post-intervention. This study also adds to the small body of literature that examines sanitation-related stress within an urban, high-density, informal settlement population [10–12, 34].

Our results align with findings from India [16], South Africa [17], Uganda [18], and Kenya [19] that unimproved sanitation options cause psychosocial stress including disgust, shame, fear of violence, and perpetrated violence. However, in contrast to the current research, these studies do not explore whether stressors may be mitigated given a shared improved sanitation option in an informal slum environment.

The research team expected and our findings confirmed that improved shared latrines decreased sanitation-related stress for the majority of users compared to traditional shared latrines. However, this reduction in stress was not uniform across all stress domains. We found significant decreases in both the frequency and severity of privacy related stress among all improved shared sanitation users. However, safety and management concerns continue to persist among some female SL and CSB users; and in some cases were worse than their traditional latrine user counterparts. Safety and management stress are exacerbated by high population density, in line with quantitative findings from urban Uganda [34]. Some women reported urinating in a bucket inside the home during the night time or asking a friend or partner to accompany them to the latrine during night to mitigate security concerns. However, essentially no SL or CSB users reported withholding defecation or practicing open defecation to mitigate stressors. In contrast, Heinjen et al.’s study in Odisha, India found that women frequently withheld defecation or practiced open defecation due to safety and privacy concerns [9].

In one of the few existing studies on improved shared sanitation and related stress in an urban area, Nelson et al., finds that women in Indonesia who share a latrine among two households have a higher relative risk of feeling safe at night compared to women sharing an improved facility among three or more households [13]. Our findings align with this conclusion: CSB users, who share with larger groups of people than even the traditional latrine users in our sample, were more likely to report feeling unsafe. Despite the fact that each latrine is designed to be used by a maximum of 20 people, compounds with larger populations found it more difficult to share their latrines and experienced more stressors than compounds with smaller populations. Tumwebaze et al.’s study on user satisfaction of shared latrines in Kampala, Uganda also found that a larger number of latrine sharers was a determinant of user dissatisfaction [34]. Additional research could investigate how to mitigate safety concerns among large groups of shared latrine users.

In urban and rural Odisha, India, Sahoo and colleagues. Found that sanitation-related stress was mediated by life stage in Odisha, India [7]. In urban Maputo, our research found few differences among girls and women at different life stages and that varying childcare and marriage responsibilities do not necessarily dictate differences in latrine use and sanitation stress. Sahoo et al. also found three domains of sanitation-related stress – environmental, social, and gender-based violence stressors. Stressors identified in our study – safety, privacy, disgust, and management – reflect many of the same underlying social and environmental processes; however, the resultant stress experience at each site resulted in unique stress domains. These findings demonstrate that experiences of sanitation-related stress among women are not static and vary based on geographical and social context. In India, for example, the primary focus of sanitation programs and sanitation interventions is the transition from open defecation to private, household-level facilities; stress related to shared resources was often focused on access to shared water supply rather than maintenance and upkeep of shared sanitation infrastructure. Sanitation-related social interactions and gendered understandings of stress, dignity, and vulnerability in one community do not necessarily translate to the global experience of impoverished women.

The research findings highlight the complex spatial realities of latrine placement and the often complex and contradictory requirements for ideal placement of shared sanitation facilities in compounds. Equidistant placement of new latrines from all households could improve perceptions of safety and access, particularly at night. Further, this will maximize privacy for residents using the latrine. However, privacy concerns also mean that latrines should not be placed in the middle of shared spaces as this can make it too easy to observe, hear, and smell defecation. Building close to the edges of the property often makes construction and access to the latrine and/or septic system easier, although locations on the periphery of compounds may increase concerns about safety. Our findings show that there is not a one-size-fits-all approach to positioning the latrine to mitigate stressors. The only way to manage such contradictory requirements is through engagement and consultation with end-users, particularly female users.

Compound fencing, compound lighting, and door locks are major determinants of perceptions of safety. We note differences in reported locks between CSB and SL users – with over twice as many CSB users reporting that their latrine doors lock compared to SL users. Due to the larger number of users, there are often more complex management structures for CSBs. This may explain the greater proportion of latrines with locks. However, we note that these complex management systems can be more difficult to maintain at the compound level. Although compound members live in close vicinity to one another, they may not be friends; disagreements are prone to occur, creating sanitation-related stressors. Taking best practices from compounds with functional latrine management systems, community based organizations or other WASH practitioners can work with community members to create cleaning schedules, guidelines for ongoing latrine maintenance, and simple accountability mechanisms to decrease neighborhood conflict, feelings of disgust, and stress from lack of management.

Compound fencing and walls and compound lighting may be beyond the scope of most sanitation interventions. The increased availability of low-cost, solar charged lights may provide an opportunity to improve safety within compounds and warrant further exploration. Integrating latrine improvements with fencing improvements may provide another incentive for compounds to invest in sanitation improvements.

## Limitations

The transferability of our findings is limited, as the research reflects the specific context of shared WSUP-constructed latrines in peri-urban slum communities in Maputo. However, many of the discussed themes may hold true for female users of shared latrines in other urban, low-income sub-Saharan settings. The current research is somewhat limited in its capacity to understand why safety perceptions may differ between SL and CSB users. A larger sample and additional investigation is necessary to determine the significance and cause of these apparent differences.

Reports of the frequency and severity of stressors should be interpreted with caution. In contrast to surveys, qualitative interviews do not follow a set sequence of questions with pre-defined answers. All data must be interpreted within the context of the broader two-way discussion between interviewer and respondent. Our classification of stressors was based on our subjective interpretation of a respondent’s answers and may not reflect the participants own lived experience. Further, some respondents may not have discussed a specific stressor because the interviewer did not ask, the conversations focused on other stressors, or for a host of other reasons. We feel our minimal quantification, however, is useful for identifying larger patterns within the data and no statistical analysis are proposed. Future survey efforts will more formally quantify stressors and experiences related to shared sanitation within this population.

## Conclusions

Shared sanitation systems are an increasingly common reality in urban environments where both resources and space are limited. Share sanitation presents specific stressors to users – specifically disgust, privacy, safety, and management related stressors. Properly managed and maintained shared improved sanitation interventions can reduce sanitation-related stress, and future shared sanitation interventions should be sensitive to the needs and requirements of users. Management structures – the focus of a forthcoming analysis of the same data – play a central role in reducing stress among users, and interventions should ensure that that appropriate attention is given to fostering and supporting inclusive management systems.

## Footnotes

1 Safe fecal management is defined as excreta that are safely disposed of in situ or transported and treated offsite.

2 Some traditional pit latrines have a concrete slab, which meets JMP’s definition of a limited improved latrine.
